# Impulsive choice in individuals with comorbid amphetamine use disorder and attention deficit-hyperactivity disorder

**DOI:** 10.1186/s12888-023-05034-x

**Published:** 2023-07-24

**Authors:** Christoffer Brynte, Lotfi Khemiri, Hannes Stenström, Maija Konstenius, Nitya-Jayaram Lindström, Johan Franck

**Affiliations:** Department of Clinical Neuroscience, Centre for Psychiatry Research, Karolinska Institutet & Stockholm Health Care Services, Stockholm County Council, Stockholm, Sweden

**Keywords:** ADHD, Substance use disorder, Impulsive behavior

## Abstract

**Background:**

Amphetamine use disorder (AMPH) and attention deficit-hyperactivity disorder (ADHD) often co-occur and are associated with poor treatment outcomes. Elevated impulsivity is a core feature in both disorders. Little is known however about the specific neurocognitive profile regarding different facets of impulsivity, and specifically impulsive choice, in comorbid populations.

**Methods:**

Three groups (ADHD + AMPH, ADHD only and healthy controls (HC)) were assessed with self-reported impulsivity and cognitive tasks of impulsive choice, operationalized as delay aversion (DA) and reflection impulsivity.

**Results:**

Twenty-nine participants with comorbid ADHD + AMPH, 25 participants with ADHD only and 116 HC completed screening, including self-rating scales, and cognitive testing. 20, 16 and 114 participants completed computerized cognitive tasks in the ADHD + AMPH group, ADHD group and HC group, respectively. The ADHD + AMPH group reported significantly higher motor, attentional and non-planning impulsiveness, and showed a significantly higher degree of impulsive choice, compared to both groups. There were no differences in task-related impulsiveness between ADHD only and HC.

**Conclusions:**

The current findings suggest that individuals with ADHD + AMPH have overall elevated levels of impulsivity compared to individuals with ADHD only. In addition, that ADHD + AMPH is specifically associated with impairments in task-related impulsive choice, which was not found in ADHD only compared to HC. The neurocognitive profile in this specific patient group may represent a need for more systematic screening within healthcare settings in order to develop effective and targeted treatment for comorbid patients.

**Trial registration:**

EudraCT, 2012–004298-20.

**Supplementary Information:**

The online version contains supplementary material available at 10.1186/s12888-023-05034-x.

## Introduction

Substance Use Disorder (SUD) and Attention Deficit/Hyperactivity Disorder (ADHD) often co-occur [1, 2] and share several overlapping neurocognitive characteristics, such as impaired disinhibition (or motor impulsivity), emotional dysregulation and elevated sensation seeking [3]. Moreover, studies have shown that having both disorders is associated with more pronounced deficits [4–6], a more severe course of illness and poorer treatment outcomes [7, 8]. Importantly, pharmacological treatments for ADHD do not seem to be as effective for individuals suffering from comorbid ADHD and SUD [9].

Elevated impulsiveness is a core feature in both ADHD and SUD, which in itself has been shown to predict relapse and poor SUD treatment outcomes [3]. A commonly held definition of impulsivity was proposed by Moeller and colleagues as “a predisposition toward rapid unplanned reactions to internal or external stimuli without regard to the negative consequences of these reactions to the impulsive individual or to others” [10]. Even though there is no consensus on the structure of the construct of impulsiveness, it is often conceptualized as a heterogenous construct with different components which contribute to the expression of impulsive behavior [11]. It has been suggested that impulsivity can be fractionated into at least three constructs that contribute to SUD and ADHD: a) Disinhibition, b) impulsive choice and c) sensation seeking [3]. These constructs may on the individual level not be associated, and the clinical importance of the interaction(s) between the three in ADHD and SUD, is largely unknown. It is therefore important to investigate how these underlying constructs of impulsiveness manifests in the context of comorbid SUD and ADHD.

The current paper has a focus on impulsive choice, which is considered to reflect impulsive behavior as a result of delayed discounting (DD) and/or reflection impulsivity. Where DD is the tendency of devaluating delayed greater rewards in favor of immediate smaller rewards, and reflection impulsivity is the tendency to not collect enough information prior to a decision. Related to, but distinguished from DD, is delay aversion (DA) which refers to the avoidance of delays as a consequence of a negative emotional response due to delay overall [12].

There has been extensive research on neurocognitive functioning in both ADHD and SUD populations during the last decades and impulsive choice have been found to be associated with both disorders [3]. Specifically, amphetamine use has consistently been found to be associated with higher levels of impulsivity [13], although the causal relationship is yet largely unknown. Importantly, stimulant treatment of ADHD, with well-documented short-term enhancing effects on cognition, has not been found to have adverse long-term effects on cognition [14].

Only a few studies regarding impulsive choice have been conducted in a controlled human laboratory setting, in ADHD/SUD comorbid populations. One study found steeper DD in individuals with comorbid cocaine use disorder (CUD) and ADHD, compared to individuals with ADHD only [5]. This is consistent with findings in two recent studies investigating comorbid individuals with ADHD, substance misuse and cannabis use disorders, respectively [15, 16]. Similarly, another study found steeper DD in comorbid CUD/ADHD compared to individuals with CUD only [17]. These findings suggest that impulsive choice is more pronounced in comorbid populations. However, only one study utilized a task of reflection impulsivity, and DA has to the best of our knowledge not been directly investigated in comorbid ADHD/SUD individuals. In summary, it remains unclear how different aspects of impulsive choice manifests in comorbid ADHD/SUD individuals, and how these manifests across different SUDs, which has implications on how to best treat comorbid individuals. To the best of our knowledge no previous study has investigated impulsive choice in a human laboratory setting in individuals with comorbid ADHD + AMPH. Notably, amphetamine use is much more common in Sweden compared to methamphetamine use [18].

The overall aim of the current study was to examine different aspects of impulsive choice in individuals with comorbid AMPH + ADHD compared to ADHD only. We hypothesized that individuals with AMPH + ADHD would have higher 1) DA and 2) impaired reflection impulsivity, as measured by Cambridge Gambling Task (CGT) [19] and Information Sampling TASK (IST) [20], respectively. Furthermore, we hypothesized that individual with AMPH + ADHD score higher on self-reported impulsive behavior as measured by the Barratt Impulsiveness Scale (BIS-11) [21].

## Materials and methods

### Participants

Adult (> 18 years old) males and females were recruited through public advertising and active recruitment from substance dependence treatment services in the Stockholm region, from August 2015 to December 2019. The patients were recruited to a clinical trial (unpublished)investigating the dose-dependent response of methylphenidate in comorbid SUD/AUD (Trial registration number: EudraCT, 2012–004298-20, 14/11/2014). The study was approved by the Swedish Ethical Review Authority (DNR 2012/1407–31/1) and participants provided their consent following oral and written information.

Three groups were recruited: 1) participants with comorbid AMPH/ADHD; 2) ADHD only and 3) HC. Participants were assessed for medical and psychiatric disorders by a designated study physician, including physical examination, pregnancy test, alcohol breathalyzer test, urine toxicology, blood analysis (complete blood count, liver function tests, electrolytes, proBNP, thyroid hormone levels), blood pressure, heart rate and ECG. The presence of ADHD was assessed in accordance with national guidelines including the Diagnostic Interview for ADHD in Adults (DIVA 2.0) [22], a short version of Weschler Adult Intelligence Scale [23] (a structured instrument for assessing intelligence is a national requirement in ADHD diagnostics), and interviews with family members. For participants that were already diagnosed with ADHD, the study physician assessed patient records to confirm a valid diagnostic procedure in accordance with national guidelines, including structured diagnostic instruments. Additionally, all participants were assessed with the Mini International Neuropsychiatric Interview.

Main inclusion criteria were moderate to severe AMPH and/or ADHD according to DSM-5 [24]. For AMPH participants, they were required to have been abstinent from amphetamine for a minimum of 7, and a maximum of 90 days, before inclusion as judged by self-report and the absence of amphetamine in supervised urine test at screening and inclusion. I.e., the acceptable range for abstinence from amphetamine was 7 to 90 days.

Main exclusion criteria were presence of severe somatic disorder, pregnancy, other major psychiatric disorders (such as schizophrenia, bipolar disorder, major depression as assessed with the Mini International Neuropsychiatric Interview), severe SUD except nicotine- and amphetamine use disorder, use of psychoactive substances during the last seven days, or any pharmacological ADHD treatment during the last 14 days, preceding enrollment. Additionally, participants in the ADHD only group and HC, were excluded if they had a history of SUD (excluding nicotine).

## Instruments and procedures

Participants in the ADHD + AMPH and ADHD groups were assessed with self-rating scales, including BIS-11, at screening, regardless of current substance use and/or pharmacological treatment. Cognitive tasks were conducted at a later date to ensure the mandatory wash-out period of 7 days from illicit substances and 14 days from pharmacological ADHD treatment.

### Screening tools

Participants were assessed for substance use via the Time-Line Follow Back interview (TLFB) [25] and a study-specific modified version of Lifetime Drinking History Interview (LDH) for amphetamine use. To assess presence of depressive symptoms, the Montgomery Åsberg Depression Rating Scale (MADRS) [26] was utilized. Sociodemographic data was collected through a questionnaire, specific for the current study.

### Cognitive assessments

CGT and IST from the Cambridge Neuropsychological Test Automated Battery (CANTAB®), were administered using a touch-screen tablet PC (MOTION J3500-i7B) and a press pad from Cambridge Cognition Ltd.

### Barratt Impulsiveness Scale (BIS)

Self-reported impulsive behavior was assessed via the Barratt Impulsiveness Scale, a widely used self-report measure of trait impulsivity. In this study, we utilized a validated Swedish translation of the BIS-11. The main subscales measure attentional, motor and non-planning impulsiveness.

### Information Sampling Task (IST)

IST is a task of reflection impulsivity with an administration time of approximately 15 min. The test is constructed as a game in which the participant is presented with a 5 × 5 grid of gray boxes and two larger colored panels below this grid. The purpose of the game is to correctly guess which color the majority of the boxes have under the gray layer (yellow or green). Participants are free to show the underlying color of the boxes one by one. In the Fixed Win condition, no penalty is given for showing the color of the boxes and 100 points is given for a correct guess. In the Decreasing Win condition, the number of points that the participant can win is 250 but decreases by 10 points for every box that the participant shows the color of. There is a fixed time between each trial, meaning that participants will have to wait longer before next trial if they guess quickly, i.e. there is no gain in the time it takes to complete the task by guessing quickly. The key outcome measures of the IST reflect the tendency, or lack thereof, to collect information prior to making decisions.

### Cambridge gamble task

The Cambridge Gambling Task is a computerized test aimed at evaluating quality of decision-making and risk taking. The participant is presented with ten boxes which are either red or blue. The ratio of red:blue boxes varies between trials. The participant is informed that a token is hidden in one of the boxes and that they must make a guess whether the token is in a red or a blue box. The participant then has to place points stake on their guess. If the guess is correct points get added to the participant’s total score and points are deducted if the guess is wrong. The participant is free to choose from a range of bets which are presented sequentially, ranging from a small to a large percentage of the participants current score. The game is performed in two conditions. In one condition of the test, the bets are presented in ascending order with the bets getting larger and larger. In the other condition the bets are getting smaller and smaller.

Key outcome measures of CGT reflect the tendency to adjust decisions according to risk and DA.

### Statistical analysis

Data was processed and analyzed using R Studio software, version 2021.09.0. Further details on R packages and code are found in supplementary material. Shapiro–Wilk test was used to assess normality and Levene's test to assess homogeneity of variances. Outliers were assessed visually via box-plot. A one-way analysis of variance (ANOVA) was used to investigate main effect of group (independent variable) on the different cognitive outcomes and age (dependent variables). Significant main effects of groups were followed by a post hoc Fischer's protected least significance difference, and a Tukey's Honest Significant Difference test (for ANOVA) or Bonferroni correction (for Kruskal Wallis) for the main and sensitivity analysis, respectively. In the case where test assumptions were violated, the robustness of any results were assessed with complimentary non-parametric statistical testing (Kruskal Wallis H-test) and/or Welch ANOVA. Effect size for the main effects of group is reported as eta squared. Exploratory analysis was performed, adjusting for covariates such as gender and age, and to investigate correlations between study measures. Categorical variables were compared utilizing chi-square test.

## Results

A total of 170 participants were recruited and divided into three different groups. Twenty-nine participants with comorbid ADHD + AMPH, 25 participants with ADHD only and 116 HC completed screening, including BIS-11, and cognitive testing. 20, 16 and 114 participants completed computerized cognitive tasks in the ADHD/AMPH group, ADHD group and HC group, respectively. Sociodemographic and clinical data are presented in Tables 1 and 2.

**Table 1:** Sociodemographic data. Data presented as mean(standard deviation), unless otherwise stated

	**AMPH/ADHD (*n* = 29)**	**ADHD (*n* = 25)**	**HC (*n* = 116)**
Age	40.7 (10.3)	36.9 (11.4)	45.9 (12.2)^a^
Sex	12 Male (41.4%) + 17 Female (58.6%)	12 Male (48.0%) + 13 Female (52.0%)	52 Male (44.8%) + 64 Female (55.2%)
Years in school			
< 9 years	-	-	0.9%
< 12 years	41.2%^a^	16.0%^a^	3.4%^a^
< 15 years	34.5%	40.0%	41.4%
≥ 15 years	0.0%^a^	40.0%	53.4%
Unknown/Missing	24.1%	4.0%	0.9%
Income through paid work			
No	62.1%^a^	16.0%	12.9%
Yes	10.3%^a^	60.0%	86.2%
Missing	27.6%	24.0%	0.9%
Housing			
Homeless	20.7%^a^	-	1.7%
Apartment/House	24.1%^a^	56.0%	87.1%
Room	10.3%	8.0%	5.2%
Shelter/Social services	27.6%^a^	-	0.9%
Unknown/Missing	17.2%	36.0%	5.2%
Social status			
Never lived with partner/spouse	31.0%	32.0%	28.4%
Married	6.9%	12.0%	33.6%
Living with someone	10.3%	20.0%	19.8%
Divorced/Separated	17.2%	4.0%	14.7%
Unknown/Missing	34.5%	32.0%	3.4%

A one-way ANOVA was conducted with post-hoc pairwise comparisons for continuous variables (age) and chi-square test for categorical variables

^a^Significant difference compared to the other two groups, respectively

**Table 2:** Clinical data. Data are presented as mean (standard deviation), unless otherwise stated

	**AMPH/ADHD** **(*n* = 29)**	**ADHD** **(*n* = 25)**	**HC** **(*n* = 116)**
Previous depression	41.4%^c^	64.0%^b^	7.8%^a^
Previous episode with psychotic symptoms due to stimulant use	20.7%^a^	-	-
Previous agoraphobia	10.3%^c^	0.0%	0.0%
Previous panic disorder	20.7%^c^	16.0%^b^	0.0%^a^
Previous posttraumatic stress syndrome	3.4%	4.0%	0.0%
Previous suicide attempt	10.3%^b^	8.0%	0.0%^b^
Previous eating disorder	0.0%	8.0%	2.6%
Borderline personality disorder	3.4%	0.0%	0.0%
Antisocial personality disorder	20.7%^a^	0.0%	0.0%
Present social anxiety	10.3%^c^	4.0%	0.0%
Present agoraphobia	6.9%	0.0%	0.0%
Bipolar II disorder	0.0%	4.0%	0.0%
Autism spectrum disorder	3.5%	12.0%^b^	0.0%^b^
Daily nicotine use			
Yes	93.1%^a^	28.0%^a^	8.6%^a^
Missing	3.4%	-	0.9%
Years with regular amphetamine use	13.5 (10.0)	-	-
Continuous days without substance use prior to cognitive testing			
Mean (SD)	23.6 (17.4)	-	-
Minimum	7		
Maximum	67		
Route of administration (amphetamine)			
Intravenously	44.8%		
Oral/Snorting	24.1%		
Missing	31.0%		
MADRS	16.9 (8.55)^a^	11.1 (8.29)^a^	3.54 (4.01)^a^

*MADRS* Montgomery Åsberg Depression Rating Scale. Due to violations of test assumptions a non-parametric test and post hoc test was utilized

^a^Significant post hoc differences between the other two groups, respectively

^b^Significant difference between ADHD only and HC

^c^Significant difference between AMPH/ADHD and HC

## Cognitive assessment

The ADHD + AMPH group presented with significantly higher self-reported impulsive behavior and showed greater impaired task-related delay aversion and reflection impulsivity compared to ADHD only and HC. Results from cognitive assessments are presented in Table 3.

**Table 3:** Results from self-reported impulsivity, as measured by Barratt Impulsivity Scale (BIS) and the cognitive tasks Cambridge Gambling Task (CGT), and Information Sampling Task (IST)), in individuals with comorbid amphetamine use disorder and ADHD (AMPH/ADHD), ADHD only and healthy controls (HC). Data are presented as mean ± standard deviation

	**AMPH/ADHD** **(*n* = 29)**	**ADHD** **(*n* = 25)**	**HC** **(*n* = 116)**	**ANOVA** **η2**	**Post hoc analysis**^**a**^
BIS total	85.6 ± 11.6	73.1 ± 13.4	55.4 ± 8.32	[F(2, 158) = 116.2, *p* < 0.01] η2 = 0.60	ADHD + AMPH > ADHD > HC
BIS motor	29.4 ± 5.9	25.5 ± 5.1	20.1 ± 3.6	[F(2, 161) = 59.0, *p* < 0.01] η2 = 0.42	ADHD + AMPH > ADHD > HC
BIS attention	22.9 ± 3.6	20.4 ± 3.9	12.9 ± 2.8	[F(2, 161) = 144.6, *p* < 0.01] η2 = 0.64	ADHD + AMPH > ADHD > HC
BIS non-planning	33.2 ± 4.5	27.2 ± 6.8	22.5 ± 4.6	[F(2, 159) = 53.6, *p* < 0.01] η2 = 0.40	ADHD + AMPH > ADHD > HC
CGT					
Delay aversion	0.315 ± 0.207	0.165 ± 0.182	0.157 ± 0.178	[F(2, 147) = 6.47, *p* < 0.01] η2 = 0.08	ADHD + AMPH > (ADHD = HC)
Risk adjustment	0.317 ± 1.42	1.39 ± 1.13	1.46 ± 0.909	[F(2, 147) = 10.84, *p* < 0.01] η2 = 0.13	ADHD + AMPH < (ADHD = HC)
IST					
Boxes opened, DW	5.84 ± 3.09	9.16 ± 3.24	10.0 ± 4.98	[F(2, 135) = 6.9, *p* < 0.01] η2 = 0.09	ADHD + AMPH < (ADHD = HC)
Boxes opened, FW	8.78 ± 4.60	14.6 ± 5.08	17.0 ± 5.67	[F(2, 135) = 19.2, *p* < 0.01] η2 = 0.22	ADHD + AMPH < (ADHD = HC)
P (correct), DW	0.630 ± 0.086	0.731 ± 0.080	0.736 ± 0.107	[F(2, 135) = 9.24, *p* < 0.01] η2 = 0.12	ADHD + AMPH < (ADHD = HC)
P (correct), FW	0.668 ± 0.104	0.818 ± 0.112	0.852 ± 0.114	[F(2, 135) = 22.3, *p* < 0.01] η2 = 0.25	ADHD + AMPH < (ADHD = HC)

^a^Greater/lesser than symbols indicates post hoc significant difference and it’s direction. The equal symbol indicates no significant post hoc difference. There was also a significant difference between ADHD + AMPH and HC on the CGT outcome “Quality of Decision Making” (not shown in table), but not between the other groups. There were no differences between groups on the CGT outcomes “Risk Taking” and “Overall Proportion Bet” (not shown in table)

For many of the outcomes data was not normally distributed or homogeneity of variance was not met, or there were some outliers. However, when performing the non-parametric tests described above the results were always identical (or very similar), with one exception, to the main ANOVA analysis and therefore we report only the ANOVA results to facilitate the interpretation of the results. For the outcome “Quality of Decision Making” in the CGT, the non-parametric results are presented. Sensitivity analysis with adjustment for multiple comparisons yielded similar results, which did not affect the overall conclusions (the full analysis including non-parametric test results are presented in the supplementary material).

## Cambridge gambling task

### Delay aversion

The comorbid (ADHD + AMPH) group demonstrated a significantly higher DA score compared to individuals with ADHD only (*p* = 0.02) and HC (*p* < 0.01), indicating that the ADHD/AMPH group were more unwilling and/or unable to wait prior to making decisions. There was no significant difference between ADHD and HC (*p* = 0.86).

### Risk adjustment

The ADHD + AMPH group had a significantly lower score of Risk Adjustment, compared to the ADHD group (*p* < 0.01) and HC (*p* < 0.01), indicating less adjustment of betting behavior according to risk. There was no significant difference between ADHD and HC (*p* = 0.79).

### Risk taking, quality of decision making and overall proportion bet

There was no significant difference between groups on Risk Taking (*p* = 0.75). The ADHD + AMPH group had a significantly lower Quality of Decision Making compared to the HC group (*p* = 0.004), while there were no significant differences between ADHD + AMPH and the ADHD only group, nor between ADHD only and HC. There were no significant differences between groups on Overall Proportion Bet (*p* = 0.95).

## Information sampling task

### Mean number of boxes opened per trial – decreasing win condition

Compared to the ADHD only (*p* = 0.03) group and HC (*p* < 0.01), the ADHD + AMPH group opened significantly fewer boxes prior to their decision in the decreasing win condition, indicating impairment in reflection impulsivity. There was no significant difference between ADHD and HC (*p* = 0.50).

### Mean number of boxes opened per trial—fixed win condition

Compared to the ADHD only (*p* < 0.01) group and HC (*p* < 0.01), the ADHD + AMPH group opened significantly fewer boxes prior to their decision in the fixed win condition, indicating impairment in reflection impulsivity. There was no significant difference between ADHD and HC (*p* = 0.10).

### Mean P (correct) at point of decision—decreasing win condition

The ADHD + AMPH group had a significantly lower mean probability of making a correct choice at the point of decision, in the decreasing win condition, compared to the ADHD only group (*p* < 0.01) and HC (*p* < 0.01), indicating impairment in reflection impulsivity. There was no significant difference between ADHD and HC (*p* = 0.84).

### Mean P (correct) at point of decision—fixed win condition

The ADHD + AMPH group had a significantly lower mean probability of making a correct choice at the point of decision, in the fixed win condition, compared to the ADHD only group (*p* < 0.01) and HC (*p* < 0.01), indicating impairment in reflection impulsivity. There was no significant difference between ADHD and HC (*p* = 0.26).

### Barratt Impulsiveness Scale (BIS)

The ADHD + AMPH group had a significantly higher total mean score of self-reported trait impulsiveness compared to the ADHD only group (*p* < 0.01) and HC (*p* < 0.01). The ADHD group had a significantly higher mean score than the HC (*p* < 0.01).

#### BIS Motor

The ADHD + AMPH group scored significantly higher on the motor impulsivity BIS-11 subscale compared to the ADHD only group (*p* < 0.01) and HC (*p* < 0.01). The ADHD group had a significantly higher mean score than the HC (*p* < 0.01).

#### BIS Attentional

The ADHD + AMPH group scored significantly higher on the attentional impulsivity BIS-11 subscale compared to the ADHD only group (*p* < 0.01) and HC (*p* < 0.01). The ADHD group had a significantly higher mean score than the HC (*p* < 0.01).

#### BIS Non-planning

The ADHD + AMPH group scored significantly higher on the non-planning impulsivity BIS-11 subscale compared to the ADHD only group (*p* < 0.01) and HC (*p* < 0.01). The ADHD group had a significantly higher mean score than the HC (*p* < 0.01).

### Covariates and correlations

Each one-way ANOVA above remained statistically significant when controlling for sex and age. As shown in Table 4, there were no significant correlations between cognitive measures and number of years with amphetamine use, nor with number of days since last use of amphetamine. Age was negatively correlated with the BIS total score, and specifically with attention and motor impulsivity, but not with non-planning.

**Table 4:** Correlation matrix

	**BIS Total**	**BIS** **Attent**	**BIS** **Motor**	**BIS** **Nonpl**	**CGT DA**	**CGT** **Risk**	**IST** **N.FW**	**IST** **p.FW**	**Amph. years**	**Amph** **Last intake**
BIS Total	1.00									
BIS Attent	0.89*	1.00								
BIS Motor	0.88*	0.70*	1.00							
BIS Nonpl	0.90*	0.71*	0.67*	1.00						
CGT DA	0.19*	0.16	0.07	0.23*	1.00					
CGT Risk	-0.28*	-0.16*	-0.27*	-0.29*	-0.35*	1.00				
IST N.FW	-0.41*	-0.32*	-0.35*	-0.38*	-0.22*	0.33*	1.00			
IST p.FW	-0.43*	-0.33*	-0.36*	-0.44*	-0.28*	0.40*	0.87*	1.00		
Amph. years	-0.21	-0.05	-0.33	-0.04	-0.09	0.31	-0.04	-0.38	1.00	
Amph intake	-0.16	-0.02	-0.06	-0.36	-0.32	0.15	0.04	-0.06	-0.22	1.00
Age	-0.23*	-0.34*	-0.19*	-0.12	0.02	-0.18*	0.02	-0.01	0.66*	-0.09

Pearson correlation matrix. *BIS* = Barratt Impulsivity Scale. Attent., Motor, Nonpl., refers to the three subscales of BIS (attentional, motor, non-planning). CGT = Cambridge Gambling Task. ‘DA’ and ‘Risk’, refer to CGT outcome measures delay aversion and risk-adjustment. IST = Information Sampling Task. ‘N.FW’ and ‘p.FW’ refer to IST outcome measures of number of opened boxes and probability of making correct choice at time of decision. ‘Amph.years’ = Number of years with regular amphetamine use. ‘Amph.intake’ = Number of continuous days of abstinence

*Indicates a significant correlation

## Discussion

The present study aimed to investigate self-rated impulsivity and impulsive choice, operationalized as delay aversion (DA) and reflection impulsivity, in individuals with comorbid ADHD + AMPH, compared to individuals with ADHD only and HC. The ADHD + AMPH group demonstrated significantly higher levels of impulsiveness compared to the ADHD only group and HC’s, both in regard to self-reported impulsivity and as measured by cognitive tasks. Although the ADHD only group had significantly higher self-reported impulsiveness, compared to HC, there were no differences between the two control groups on task-performance.

Specifically, the ADHD + AMPH group, compared to the control groups, showed more pronounced delay aversion (DA), i.e., they were significantly less able/willing to withstand delay prior to making decisions, and showed a significantly smaller tendency towards risk adjustment (i.e., to adjust decisions according to risk). Moreover, they collected significantly less information prior to decisions, indicating impaired reflection impulsivity. These findings are in line with previous research on individuals with comorbid cocaine use disorder and ADHD [5, 6]. However, the present study is the first, to the best of our knowledge, to demonstrate through cognitive tasks, that individuals with ADHD + AMPH have significantly higher levels of different aspects of impulsive choice, compared to individuals with ADHD only, and specifically more pronounced impairments in DA, risk adjustment and reflection impulsivity. Furthermore, our results suggest that ADHD + AMPH is significantly associated with more impairments in self-reported motor, attentional and non-planning impulsiveness, compared to ADHD only.

A previous study that investigated impulsiveness in CUD/ADHD, found steeper delay discounting (DD) in the comorbid group, compared to ADHD only, but found no significant difference on any of the three subscales of BIS-11 [5]. This is discrepant with findings of the current study, where the ADHD + AMPH group scored significantly higher on all three subscales. Similarly, another study found a significant difference in BIS-11 motor impulsivity, in CUD/ADHD, but no differences in the other two subscales [6]. Besides the obvious difference, where the current study investigated individuals with AMPH, these differences might be explained by differences in SUD severity, where the current study population consists of patients with severe SUD with greater psychosocial problems. The majority administered amphetamine intravenously, and half were either homeless or had temporary housing through social services and had on average a history of amphetamine use of more than a decade. Moreover, individuals with AMPH only, have previously been shown to score high on BIS-11 [21]. Another important difference in the current sample, compared to the sample in Crunelle’s study [5], is that the average number of days of abstinence upon cognitive testing is much lower (mean 23.6 compared to 677 days (83.8 days in Miguel et.al. [6])), although years of stimulant use are comparable (mean 13.5 compared to 12.3 years (unknown in Miguel et.al.’s study)). Interestingly, a systematic review found that some cognitive impairments may be masked in the acute phase of stimulant (cocaine) abstinence, and more pronounced in the intermediate phase (weeks to months). Importantly, there was no evidence of this in regard to impulsivity [27]. The explorative analysis found no correlation between days of abstinence and/or years with substance use, with any of the cognitive measures. The latter should however be interpreted with caution, given the small sample size, the study design and the relatively small differences in duration of abstinence.

The study found no evidence supporting any differences in performance on tasks of DA, risk adjustment and reflection impulsivity between the ADHD only group and HC. This is in accordance with the findings of two previous studies [5, 15], demonstrating impairments in impulsive choice, operationalized as DD, between CUD/ADHD, and substance misuse + ADHD, but not between ADHD only and HC. However, the current cross-sectional design does not allow for inference about the nature of this relationship. Interestingly, another study found that individuals with CUD/ADHD had impaired DD, compared to CUD only and HC, whereas individuals with CUD only did not [17]. Taken together, the current findings support the hypothesis that impulsive choice is uniquely impaired in individuals with comorbid stimulant use disorder and ADHD. Moreover, this is the first study to demonstrate that DA and reflection impulsivity is significantly impaired in ADHD + AMPH compared to ADHD only.

### Study strengths and limitations

There are some important limitations in the current study, of which the main one being the small sample size. Another limitation is that both the ADHD + AMPH and ADHD only groups, respectively, were recruited from participants in a clinical trial that excluded several other comorbidities and required the participants to be drug negative. This limits the external validity of the findings, and they may therefore not be representative of a broader clinical population. For instance, individuals with more severe symptoms, and other concurrent SUD,’s might not have been able to succeed with the mandatory detoxification prior to enrollment. On the other hand, the exclusion of patients with other major psychiatric comorbidities and/or current use of psychoactive substances could be viewed as a strength as it, might have confounded the results, and thus adds to the internal validity. Additionally, detailed life-time data on other illicit substances (than amphetamine) was not collected, which theoretically might have confounded results. On the other hand, no participants fulfilled DSM V criteria for any other moderate to severe SUD, other than amphetamine use disorder, during the 12 months preceding inclusion. Significantly higher degree of depressive symptoms in the ADHD groups, and the significant differences in nicotine use and sociodemographic data, might also have confounded results. Specifically, nicotine has been found to both be an enhancer of some aspects of cognition short term, while heavy smoking is associated with impairment [28]. Another important strength is that all groups had similar numbers of both sexes, further adding to the generalizability of these findings.

Theoretically, there may also be an overlap between performance on the two tasks utilized. Whereas IST is operationalized to measure reflection impulsivity, and CGT to reflect delay aversion, one cannot rule out that delay aversion to some extent also influences the performance on IST. On the other hand, the IST utilized is designed in such a way that there is no gain in delay by choosing quickly (i.e., there was a fixed time between each trial). In addition, it should be noted that even though impulsivity is viewed as a multidimensional construct, there is currently no consensus on the underlying dimensions of impulsivity. On the other hand, impulsive choice specifically, is by many authors viewed as a separate dimension of impulsivity [3, 11]. Moreover, while we found that AMPH + ADHD scored higher on BIS-11 and performed worse on CGT and IST compared HC, there was only a significant difference on BIS-11 between ADHD only and HC. Task-related impulsivity in general have been found to only correlate with self-reported symptoms of impulsivity to a low degree [29], and it is suggested that these modalities capture different dimensions of cognitive functioning. However, both modalities have proven to be important to describe psychiatric populations [11]. These differences in modalities may in part explain that we found significant differences in self-reported trait impulsivity between ADHD and HC, but no significant difference on task-related impulsive choice between these two groups. It should also be noted that all three groups consisted of middle-aged adults, which is important since impulsivity changes over the lifespan, where adolescence is associated with a higher degree of impulsivity [30]. This may also contribute to our findings and the discrepancy with previous research that suggest that there is a moderate association between task-related impulsive choice and ADHD (compared to HC) [31]. However, our findings are in accordance with previous studies with similar populations, where no significant differences in task-related impulsive choice was found between ADHD and HC [5].

Previous research suggests that impairments in impulsive choice may contribute to the development of SUD and vice versa [11], however, the present study design does not allow for causal inference. Furthermore, one cannot conclude whether impulsive choice is specifically elevated in comorbid ADHD + AMPH compared to AMPH only. A previous study found a significant difference between comorbid CUD/ADHD in DD, but not reflection impulsivity, compared to CUD only [17]. Future studies should include all three groups to further investigate the role of ADHD on impulsive choice in AMPH individuals compared to AMPH alone. Additionally, such a study should include tasks that measures different aspects of impulsive choice, including DD which was not investigated in the current study.

The findings in this study have important clinical implications and possibly contribute to the understanding of the observed weaker pharmacological effect on ADHD symptoms in comorbid populations [9]. Although a deeper discussion on this topic, including possible tolerance for stimulant treatment and the observed positive dose-dependent treatment effect in ADHD + AMPH individuals [32, 33], is outside the scope of the current paper, these findings support the hypothesis that comorbid ADHD + AMPH populations present with more pronounced neurocognitive impairments compared to ADHD only. Specifically, the results suggest that ADHD + AMPH individuals constitutes a subgroup of ADHD individuals with deficits in additional facets of impulsivity. A tendency for impulsive choices is arguably, not only important in the initiation and maintenance of SUD, but also to retention in, and success of treatment programs overall [34].

## Conclusions

The current findings suggest that individuals with ADHD + AMPH have overall elevated levels of impulsivity compared to individuals with ADHD only. In addition, that ADHD + AMPH is specifically associated with impairments in task-related impulsive choice, which was not found in ADHD only compared to HC. The neurocognitive profile in this specific patient group may represent a need for more systematic screening within healthcare settings in order to develop effective and targeted treatment for comorbid patients.

## Supplementary Information

**Additional file 1.**

## Data Availability

The datasets used and/or analysed during the current study are available from the corresponding author on reasonable request.

## Abbreviations

ADHD: Attention Deficit Hyperactivity Disorder
SUD: Substance use disorder
AMPH: Amphetamine
CUD: Cocaine use disorder
HC: Healthy control
CGT: Cambridge Gambling Task
IST: Information Sampling Task
BIS: Barrat impulsiveness scale
TLFB: Time-Line Follow-Back
DA: Delay aversion
MADRS: Montgomery Åsberg Depression Rating Scale
