# Supplementary material for: Impulsive choice in individuals with comorbid amphetamine use disorder and attention deficit-hyperactivity disorder
Source: BMC Psychiatry. 2023 Jul 24;23:537. doi: 10.1186/s12888-023-05034-x (PMC10367266; doi:10.1186/s12888-023-05034-x)
Supplement: Supplementary file 1 — Additional file 1. [file 12888_2023_5034_MOESM1_ESM.docx]

**Supplementary**

**Results From Testing of Assumptions of Statistical Tests and Sensitivity Analysis**

**Barrat Impulsiveness Scale -11**

*Total score*

There were no outliers. Data was normally distributed (p = 0.33), but the assumption of equal variance was not met (p< .001).

*BIS non-planning*

There were no outliers and data was normally distributed. The assumption of equal variance was not met (p = 0.027).

*BIS motor*

There were 3 outliers in the HC group. Data was not normally distributed with outliers kept and the assumption of equal variance was not met (p<0.5). With outliers removed data was normally distributed but did not meet the assumption of equal variance.

*BIS Attentional*

There were 5 outliers in the HC group. With or without outliers data was normally distributed. With outliers kept the assumption of equal variance was not met (p<0.5), but was met with outliers removed. Since data analysis with outliers removed, and welch ANOVA, yielded similar results as the one-way ANOVA with the outliers kept, the latter is presented.

**Cambridge Gambling Task**

*CGT Delay aversion*

There were 4 outliers, 1 in the AMPH group and 3 in the HC group. Data was not normally distributed (p< .05). Since Kruskal-Wallis test with post hoc Dunn’s test, and one-way ANOVA with the outliers removed, yielded similar results as the one-way ANOVA with the outliers kept, the presented data is from the one-way ANOVA with outliers kept.

*CGT Risk Adjustment*

There were 2 outliers in the AMPH group. Data was normally distributed, and the assumption of equal variance was met. Since Kruskal-Wallis test with post hoc Dunn’s test, and one-way ANOVA with the outliers removed, yielded similar results as the one-way ANOVA with the outliers kept, the presented data is from the one-way ANOVA with the outliers kept.

*CGT Risk Taking*

There was one outlier in the HC group. Data was normally distributed while the assumption of equal variance was not met. Since one-way ANOVA with the outliers removed, yielded similar results as the one-way ANOVA with the outliers kept, and Welch ANOVA with/without ouliers, yielded similar results as the one-way ANOVA with the outliers kept, the presented data is from the one-way ANOVA with the outliers kept.

*CGT Quality of Decision Making*

There was eleven outliers in the HC group. Data was not normally distributed and the assumption of equal variance was not met. There was a significant difference between ADHD+AMPH and HC with or without outliers, and regardless of what statistical test was utilized these results were robust. However, there was different conclusions when comparing ADHD and HC, and ADHD+AMPH and ADHD, depending on whether outliers were kept or not, or whether parametric and non-parametric tests was used. Thus, results from the non-parametric test are presented. See also supplementary table 1.

*CGT Overall Proportion Bet*

There was one outlier in the HC group. With or without the outlier, data was normally distributed and the assumption of equal variance was met. The one-way ANOVA with post hoc pairwise comparison was identical with or without the outlier. Thus, the presented data is with the outlier kept.

**Information Sampling Task**

*Mean number of boxes opened per trial – decreasing win condition*

There were no outliers and the assumption of equal variance was met. Data was not normally distributed (p< .05). Since Kruskal-Wallis test with post hoc Dunn’s test yielded similar results, the presented data is from the one-way ANOVA.

*Mean number of boxes opened per trial - fixed win condition*

There were no outliers and the assumption of equal variance was met. Data was not normally distributed (p< .05). Since Kruskal-Wallis test with post hoc Dunn’s test yielded similar results, the presented data is from the one-way ANOVA.

*Mean P (correct) at point of decision - decreasing win condition*

There were 5 outliers in the HC group. The assumption of equal variance was met, but data was not normally distributed (p< .05). Since Kruskal-Wallis test with post hoc Dunn’s test, and one-way ANOVA with the outliers removed, yielded similar results as the one-way ANOVA with the outliers kept, the presented data is from the one-way ANOVA with the outliers kept.

*Mean P (correct) at point of decision - fixed win condition*

There was 1 outlier in the AMPH group. The assumption of equal variance was met, but data was not normally distributed (p< .05). Since Kruskal-Wallis test with post hoc Dunn’s test, and one-way ANOVA with the outliers removed, yielded similar results as the one-way ANOVA with the outliers kept, the presented data is from the one-way ANOVA with the outliers kept.

Supplementary Table 1. Complementary statistical tests were performed in the case where assumptions were violated, and the table below presents the p-values of the relevant statistical test. P-values above 0.05 are in bold.

|  | One-way  ANOVA (unadjusted) | Tukey | One-way ANOVA (without outliers) | Tukey (without outliers) | Kruskal-Wallis H test with post hoc (unadjusted) | Kruskal-Wallis H test with post hoc (Bonferroni adjustment) | Welch ANOVA with Games Howell post hoc | Welch ANOVA with Games Howell post hoc  (without outliers) |
| --- | --- | --- | --- | --- | --- | --- | --- | --- |
| **BIS total score** | | | | | | | |  |
| Main effect of group | <0.001 | - | - | - | - | - | 0.001 | - |
| AMPH+ADHD vs ADHD | <0.001 | <0.001 | - | - | - | - | <0.001 | - |
| AMPH+ADHD  vs HC | <0.001 | <0.001 | - | - | - | - | <0.001 | - |
| ADHD vs HC | <0.001 | <0.001 |  |  |  |  | <0.001 | - |
| **BIS non planning** | | | | | | | |  |
| Main effect of group | <0.001 | - | - | - | - | - | 0.001 | - |
| AMPH+ADHD vs ADHD | <0.001 | <0.001 | - | - | - | - | <0.001 | - |
| AMPH+ADHD  vs HC | <0.001 | <0.001 | - | - | - | - | <0.001 | - |
| ADHD vs HC | <0.001 | <0.001 | - | - | - | - | 0.001 | - |
| **BIS motor** | | | | | | | |  |
| Main effect of group | <0.001 | - | <0.001 | - | - | - | - | <0.001 |
| AMPH+ADHD vs ADHD | 0.001 | 0.003 | <0.001 | 0.035 | - | - | - | 0.035 |
| AMPH+ADHD  vs HC | <0.001 | <0.001 | <0.001 | <0.001 | - | - | - | <0.001 |
| ADHD vs HC | <0.001 | <0.001 | <0.001 | <0.001 | - | - | - | <0.001 |
| **BIS attentional** | | | | | | | | |
| Main effect of group | <0.001 |  | <0.001 |  |  |  | <0.001 | <0.001 |
| AMPH+ADHD vs ADHD | 0.004 | 0.012 | 0.002 | 0.006 |  |  | **0.051** | **0.051** |
| AMPH+ADHD  vs HC | <0.001 | <0.001 | <0.001 | <0.001 |  |  | <0.001 | <0.001 |
| ADHD vs HC | <0.001 | <0.001 | <0.001 | <0.001 |  |  | <0.001 | <0.001 |
| **CGT Delay Aversion** | | | | | | | | |
| Main effect of group | 0.002 |  | 0.001 |  | <0.001 |  |  |  |
| AMPH+ADHD vs ADHD | 0.015 | 0.041 | 0.025 | **0.064** | 0.025 | **0.0752** |  |  |
| AMPH+ADHD  vs HC | <0.001 | 0.001 | <0.001 | <0.001 | <0.001 | 0.002 |  |  |
| ADHD vs HC | **0.861** | **0.983** | **0.556** | **0.826** | **0.754** | **1.000** |  |  |
| **CGT Risk Taking** | | | | | | | | |
| Main effect of group | **0.753** |  | **0.671** |  |  |  | **0.845** | **0.788** |
| AMPH+ADHD vs ADHD | **0.710** | **0.932** | **0.700** | **0.927** |  |  | **0.942** | **0.942** |
| AMPH+ADHD  vs HC | **0.46** | **0.485** | **0.380** | **0.360** |  |  | **0.842** | **0.799** |
| ADHD vs HC | **0.84** | **0.823** | **0.769** | **0.717** |  |  | **0.970** | **0.937** |
| **CGT Quality of Decision Making** | | | | | | | | |
| Main effect of group | <0.001 |  | <0.001 |  | <0.001 | <0.001 | 0.013 | <0.001 |
| AMPH+ADHD vs ADHD | 0.027 | **0.069** | **0.005** | **0.013** | **0.328** | **0.979** | **0.200** | **0.200** |
| AMPH+ADHD  vs HC | <0.001 | <0.001 | <0.001 | <0.001 | <0.001 | 0.002 | 0.017 | 0.004 |
| ADHD vs HC | **0.191** | **0.390** | 0.005 | 0.015 | **0.064** | **0.192** | **0.354** | **0.060** |
| **CGT Overall Proportion Bet** | | | | | | | | |
| Main effect of group | **0.953** |  | **0.909** |  |  |  |  |  |
| AMPH+ADHD vs ADHD | **0.900** |  | **0.900** |  |  |  |  |  |
| AMPH+ADHD  vs HC | **0.880** |  | **0.800** |  |  |  |  |  |
| ADHD vs HC | **0.770** |  | **0.700** |  |  |  |  |  |
| **CGT Risk adjustment** | | | | | | | | |
| Main effect of group | <0.001 |  | 0.005 |  | <0.001 |  |  |  |
| AMPH+ADHD vs ADHD | 0.002 | 0.006 | 0.028 | **0.072** | 0.019 | **0.057** |  |  |
| AMPH+ADHD  vs HC | <0.001 | <0.001 | 0.001 | 0.003 | <0.001 | 0.002 |  |  |
| ADHD vs HC | **0.793** | **0.963** | **0.777** | **0.957** | **0.899** | **1.000** |  |  |
| **IST Mean n boxes fixed win condition** | | | | | | | | |
| Main effect of group | <0.001 |  |  |  | <0.001 |  |  |  |
| AMPH+ADHD vs ADHD | 0.002 | 0.006 |  |  | 0.012 | 0.036 |  |  |
| AMPH+ADHD  vs HC | <0.001 | <0.001 |  |  | <0.001 | <0.001 |  |  |
| ADHD vs HC | **0.098** | **0.223** |  |  | **0.116** | **0.346** |  |  |
| **IST Mean n boxes decreasing win condition** | | | | | | | | |
| Main effect of group | 0.001 |  |  |  | 0.001 |  |  |  |
| AMPH+ADHD vs ADHD | 0.033 | **0.082** |  |  | 0.021 | **0.062** |  |  |
| AMPH+ADHD  vs HC | <0.001 | <0.001 |  |  | <0.001 | 0.001 |  |  |
| ADHD vs HC | **0.497** | **0.775** |  |  | **0.744** | **1.000** |  |  |
| **IST Mean P (correct) at point of decision - decreasing win condition** | | | | | | | | |
| Main effect of group | <0.001 |  | <0.001 |  | <0.001 |  |  |  |
| AMPH+ADHD vs ADHD | 0.004 | 0.010 | 0.001 | 0.003 | 0.002 | 0.011 |  |  |
| AMPH+ADHD  vs HC | <0.001 | <0.001 | <0.001 | <0.001 | <0.001 | <0.001 |  |  |
| ADHD vs HC | **0.843** | **0.978** | **0.760** | **0.950** | **0.968** | **1.000** |  |  |
| **Mean P (correct) at point of decision - fixed win condition** | | | | | | | | |
| Main effect of group | <0.001 |  | <0.001 |  | <0.001 |  |  |  |
| AMPH+ADHD vs ADHD | <0.001 | <0.001 | <0.001 | <0.001 | 0.002 | 0.006 |  |  |
| AMPH+ADHD  vs HC | <0.001 | <0.001 | <0.001 | <0.001 | <0.001 | <0.001 |  |  |
| ADHD vs HC | **0.261** | **0.498** | **0.485** | **0.504** | **0.269** | **0.806** |  |  |

**R code**

*A brief overview of code and packages used in R*

library(tidyverse)

library(ggpubr)

library(rstatix)

library(broom)

library(dplyr)

library(car)

library(dunn.test)

group_by(data_frame, Group) %>%

summarise(

count = n(),

mean = mean(dependent_variable),

sd = sd(dependent_variable)

)

boxplot.stats(data_frame$dependent_variable)$out

aov(dependent_variable ~ Group, data = data_frame)

pairwise.t.test(data_frame$dependent_variable, data_frame$Group, p.adjust.method = "none")

pairwise.t.test(data_frame$dependent_variable, data_frame$Group, p.adjust.method = "bonferroni")

TukeyHSD(one_way_anova)

leveneTest(dependent_variable ~ Group, data = data_frame)

residuals_ <- residuals(object = one_way_anova)

shapiro.test(x = residuals_)

dunn.test(data_frame$dependent_variable, data_frame$Group,

method = "none", altp = TRUE)

dunn.test(data_frame$dependent_variable, data_frame$Group,

method = "bonferroni", altp = TRUE)

oneway.test(dependent_variable ~ Group, data = data_frame)

games_howell_test(data_frame, dependent_variable~ Group, conf.level = 0.95, detailed = FALSE)

etaSquared(one_way_anova)

aov(dependent_variable~ Group + Covariate_1 + Covariate_2, data = data_frame)

chisq.test(data_frame)

pairwise_prop_test(data_frame)
